# Real-world efficacy and safety of dupilumab in the treatment of head and neck atopic dermatitis: a retrospective cohort study

**DOI:** 10.3389/fmed.2025.1705374

**Published:** 2025-11-27

**Authors:** Zifan Li, Junsheng Peng, Ruoxi Yu, Ying Lin

**Affiliations:** 1 The Second Clinical College of Guangzhou University of Chinese Medicine, Guangzhou, China; 2 Department of Dermatology, The Second Affiliated Hospital of Guangzhou University of Chinese Medicine (Guangdong Provincial Hospital of Chinese Medicine), Guangzhou, China; 3 Guangdong Clinical Research Center for Chinese Medicine Dermatology, Guangzhou, China; 4 Guangdong Provincial Key Laboratory of Chinese Medicine for Prevention and Treatment of Refractory Chronic Diseases, Guangzhou, China

**Keywords:** atopic dermatitis, dupilumab, head and neck, biologics, efficacy, safety, quality of life

## Abstract

**Background:**

Head and neck atopic dermatitis (HNAD) is a clinically common subtype of AD. While dupilumab is widely used in AD treatment, its efficacy in HNAD is debatable.

**Objective:**

This study aimed to evaluate the real-world efficacy and safety of dupilumab in HNAD.

**Methods:**

A retrospective study included 29 individuals with HNAD (17 adults and 12 pediatric patients) who were treated with dupilumab. Primary variables, including the Eczema Area and Severity Index (EASI), the Dermatology Life Quality Index (DLQI), Investigator’s Global Assessment (IGA), Patient-Oriented Eczema Measure (POEM), Visual Analog Scale for Itch (VAS-Itch), and Visual Analog Scale for Sleep Quality (VAS-SQ), were evaluated at baseline, week 4, week 8, and week 16. Data on adverse events (AEs) were routinely collected.

**Results:**

At week 16, all assessments demonstrated improvement. Both groups exhibited significant decreases (*p* < 0.05) in efficacy indicators, with the exception of children’s DLQI, which showed a decreasing trend but did not achieve statistical significance. These findings indicate that dupilumab significantly decreased the severity of the condition and improved the overall quality of life in both adults and children.

**Conclusion:**

This real-world study revealed that dupilumab could be an effective and safe therapy for HNAD patients across both children and adults.

## 1 Introduction

Atopic dermatitis (AD) is a chronic inflammatory skin disease driven by type 2 inflammation, with prevalence rates of 15–25% in children and 3–7% in adults worldwide (1). At present, the treatment of AD includes basic moisturization, topical agents, phototherapy, systemic medications, and targeted therapies. For mild AD, moisturizers, topical corticosteroids, or calcineurin inhibitors are commonly used. For moderate-to-severe cases, phototherapy and conventional systemic immunosuppressants are utilized (1). In recent years, targeted therapies, such as dupilumab and Janus Kinase (JAK) inhibitors, have revolutionized the management of moderate-to-severe AD, demonstrating significant efficacy and improved safety profiles compared with traditional immunosuppressants (2, 3).

Head and neck atopic dermatitis (HNAD) is a distinct subtype of AD that primarily affects the seborrheic areas of the body, including the scalp, face (especially the eyelids and lips), neck, and upper trunk (4). Compared with other anatomical regions, HNAD strongly affects the quality of life and exhibits increased treatment resistance due to its intricate underlying pathophysiology (5, 6). Conventional topical treatments, including corticosteroids, should be used with caution in HNAD due to the risk of skin atrophy, telangiectasia, and purpura (7). In addition, the use of topical calcineurin inhibitors may be limited due to a burning sensation and discomfort (8). Comprehensive safety studies are essential; however, data on the use of JAK inhibitors for HNAD remain insufficient (9). Consequently, only limited therapeutic alternatives are currently available for HNAD.

As a fully human monoclonal antibody, dupilumab targets the interleukin-4 receptor alpha subunit (IL-4Rα), thereby inhibiting IL-4 and IL-13 signaling pathways. It has been approved for patients aged ≥ 6 months with moderate-to-severe AD and has shown substantial efficacy and favorable safety (10–13). In global phase III clinical trials, treatment with dupilumab in adults with AD for 16 weeks resulted in EASI-50 and EASI-75 response rates of 65–69% and 44–51%, respectively (14). In a Chinese phase III trial, the corresponding rates at week 16 were 71 and 57%, respectively (15). The efficacy observed in adolescent phase III trials was comparable to that observed in adults (16, 17). In addition, phase III trials have shown comparable efficacy of dupilumab in the head and neck regions as in other anatomical areas (18, 19). However, conflicting data exist. Several studies have shown that dupilumab does not work well for treating HNAD (20, 21). Some case reports indicate that it could cause or worsen HNAD in certain individuals, a condition known as dupilumab-associated head and neck dermatitis (DAHND) (22–24). The etiology of this paradoxical occurrence may entail Malassezia colonization (5), contact allergy (25), Demodex mite infestation (26), or immunological deviation (24, 27).

Given the inconsistent findings regarding the efficacy of dupilumab for HNAD and the lack of real-world evidence specifically focused on this phenotype, this retrospective observational study aimed to evaluate the real-world efficacy and safety of dupilumab among patients with predominantly head and neck involvement in AD.

## 2 Materials and methods

This retrospective observational cohort study was conducted at Guangdong Provincial Hospital of Chinese Medicine, China, from September 2024 to March 2025. A total of 130 patients diagnosed with AD who received dupilumab treatment and were followed up for at least 16 weeks in the Chronic Disease Management Registry were enrolled. The Chronic Disease Management Registry is a Chinese registry that contains follow-up data on the effectiveness and safety of systemic therapies for the treatment of AD. The dedicated staff of the Chronic Disease Management Registry are responsible for evaluating clinical characteristics at baseline and at follow-up visits using the Eczema Area and Severity Index (EASI), the Dermatology Life Quality Index (DLQI or CDLQI for children), Investigator’s Global Assessment (IGA), the Visual Analog Scale (VAS) for Itch (VAS-Itch), the VAS for Sleep Quality (VAS-SQ), and the Patient-Oriented Eczema Measure (POEM). They are also responsible for recording adverse events (AEs) during the entire treatment period. Our study aimed to evaluate the efficacy and safety of dupilumab in patients with HNAD, defined as those with a baseline EASI score for the head and neck regions exceeding that of other body regions and those with baseline head and neck EASI (HNEASI) scores ≥7. A critical consideration is that the head and neck regions constitute a larger proportion of the total body surface area in children ≤7 years old compared to children >7 years old. Applying different multipliers will invalidate the reliability of the HNEASI score in the children’s group. Therefore, when calculating HNEASI, we did not multiply by the multiplier to ensure the accuracy of HNEASI. Among the cohort, 29 patients met the HNAD criteria. Follow-up assessments were conducted at weeks 4, 8, and 16 with a time window (7 days) for each visit. To emphasize changes in HNAD, the study specifically monitored trends in HNEASI scores and scoring curves for specific rash characteristics in the head and neck region, including erythema, excoriation, lichenification, and induration/papulation. In addition, serum total IgE and vitamin D levels at baseline were recorded following a review of the patients’ medical records.

The patients were given weight-adjusted standard-dose dupilumab treatment in accordance with the current label-recommended doses: 200 mg every 4 weeks for body weight <15 kg, and 300 mg every 4 weeks for body weight ≥ 15 kg. Moisturizers and different topical treatments, including corticosteroids and calcineurin inhibitors, were used concurrently as supplementary therapy.

All procedures conformed to standard clinical protocols. Participants provided written informed consent for data collection under routine care, following the Declaration of Helsinki principles and institutional review board approvals.

Continuous data are presented as mean ± standard deviation (SD), and categorical data as n (%). Fisher’s exact test was used to compare the children and adult groups. Paired t-tests or Wilcoxon signed-rank tests were used to assess treatment effects within groups. Statistical significance threshold was defined as a *p*-value of < 0.05. Data analyses were performed using R 4.3.1, and visualizations were generated with GraphPad Prism 9.5.1.

## 3 Results

A total of 29 patients, 17 adults and 12 children, participated in this study. The mean age of the children’s group was 12.6 ± 5.1 years, with 66.7% female participants. The mean baseline EASI score in this group was 13.9 ± 9.83. In the adult group, the mean age was 25.8 ± 10.1 years, with 52.9% female patients. The mean baseline EASI score in the adult group was 16.0 ± 11.5 (Table 1).

**Table 1:** Patient demographics and baseline clinical characteristics in our cohort.

Characteristics	Total (*n* = 29)	Children (*n* = 12)	Adults (*n* = 17)
Age	20.3 ± 10.6	12.6 ± 5.1	25.8 ± 10.1
Sex			
Male	12 (41.4%)	4 (33.3%)	8 (47.1%)
Female	17 (58.6%)	8 (66.7%)	9 (52.9%)
Family history of atopic disease	25 (86.2%)	12 (100%)	13 (76.5%)
History of urticaria	6 (20.7%)	1 (8.33%)	5 (29.4%)
History of allergic Rhinitis	16 (55.2%)	8 (66.7%)	8 (47.1%)
History of asthma	4 (13.8%)	2 (16.7%)	2 (11.8%)
History of conjunctivitis	3 (10.3%)	0 (0.00%)	3 (17.6%)
Previous use of TCS	17 (58.6%)	8 (66.7%)	9 (52.9%)
Previous use of TCI	13 (44.8%)	7 (58.3%)	6 (35.3%)
Previous use of a PDE-4 inhibitor	10 (34.5%)	4 (33.3%)	6 (35.3%)
Previous use of immunosuppressant	2 (6.90%)	1 (8.33%)	1 (5.88%)
Previous use of a JAK inhibitor	5 (17.2%)	2 (16.7%)	3 (17.6%)
Serum total IgE (mean ± s.d.)	4,489 ± 4,456	2,581 ± 3,314	5,516 ± 4,762
Serum Vitamin D (mean ± s.d.)	58.3 ± 30.9	61.5 ± 27.4	56.0 ± 35.1
EASI (mean ± s.d.)	13.9 ± 9.83	10.8 ± 6.08	16.0 ± 11.5
HN-EASI (mean ± s.d.)	27.2 ± 16.4	20.5 ± 8.83	32.0 ± 18.9
VAS-Itch (mean ± s.d.)	5.36 ± 2.16	5.92 ± 2.35	4.94 ± 1.98
VAS-SQ (mean ± s.d.)	4.21 ± 2.70	5.25 ± 2.60	3.44 ± 2.58
IGA (mean ± s.d.)	3.03 ± 0.63	2.83 ± 0.58	3.18 ± 0.64
DLQI/CDLQI (mean ± s.d.)	14.5 ± 6.65	12.2 ± 4.05	15.8 ± 7.58
POEM (mean ± s.d.)	17.2 ± 6.31	17.9 ± 4.85	16.6 ± 7.27

TCS, topical corticosteroids; TCI, topical calcineurin inhibitors; PDE-4, phosphodiesterase-4; JAK, Janus kinase; s.d., standard deviation; EASI, Eczema Area and Severity Index; HN-EASI, Eczema Area and Severity Index in Head and Neck; VAS-Itch, Visual Analog Scale for Itch; VAS-SQ, Visual Analog Scale for Sleep Quality; IGA, Investigator’s Global Assessment; DLQI/CDLQI, Dermatology Life Quality Index/Children’s Dermatology Life Quality Index; POEM, Patient-Oriented Eczema Measure.

### 3.1 Children’s group

**Figure 1:**
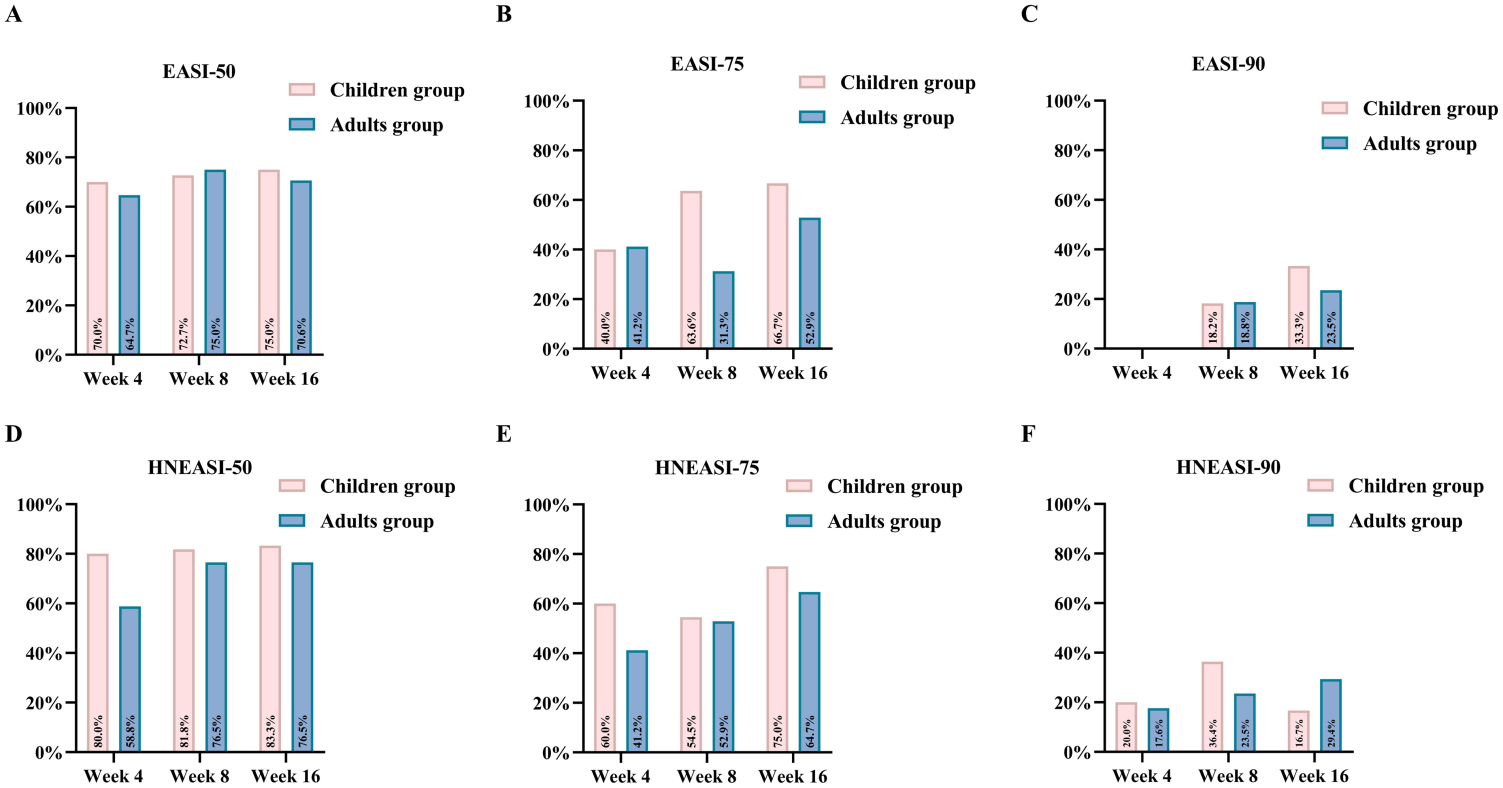
Proportion of patients achieving key efficacy endpoints from baseline during follow-up. **(A)** Proportion of patients achieving EASI-50 over time. **(B)** Proportion of patients achieving EASI-75 over time. **(C)** Proportion of patients achieving EASI-90 over time. **(D)** Proportion of patients achieving HNEASI-50 over time. **(E)** Proportion of patients achieving HNEASI-75 over time. **(F)** Proportion of patients achieving HNEASI-90 over time.

**Figure 2:**
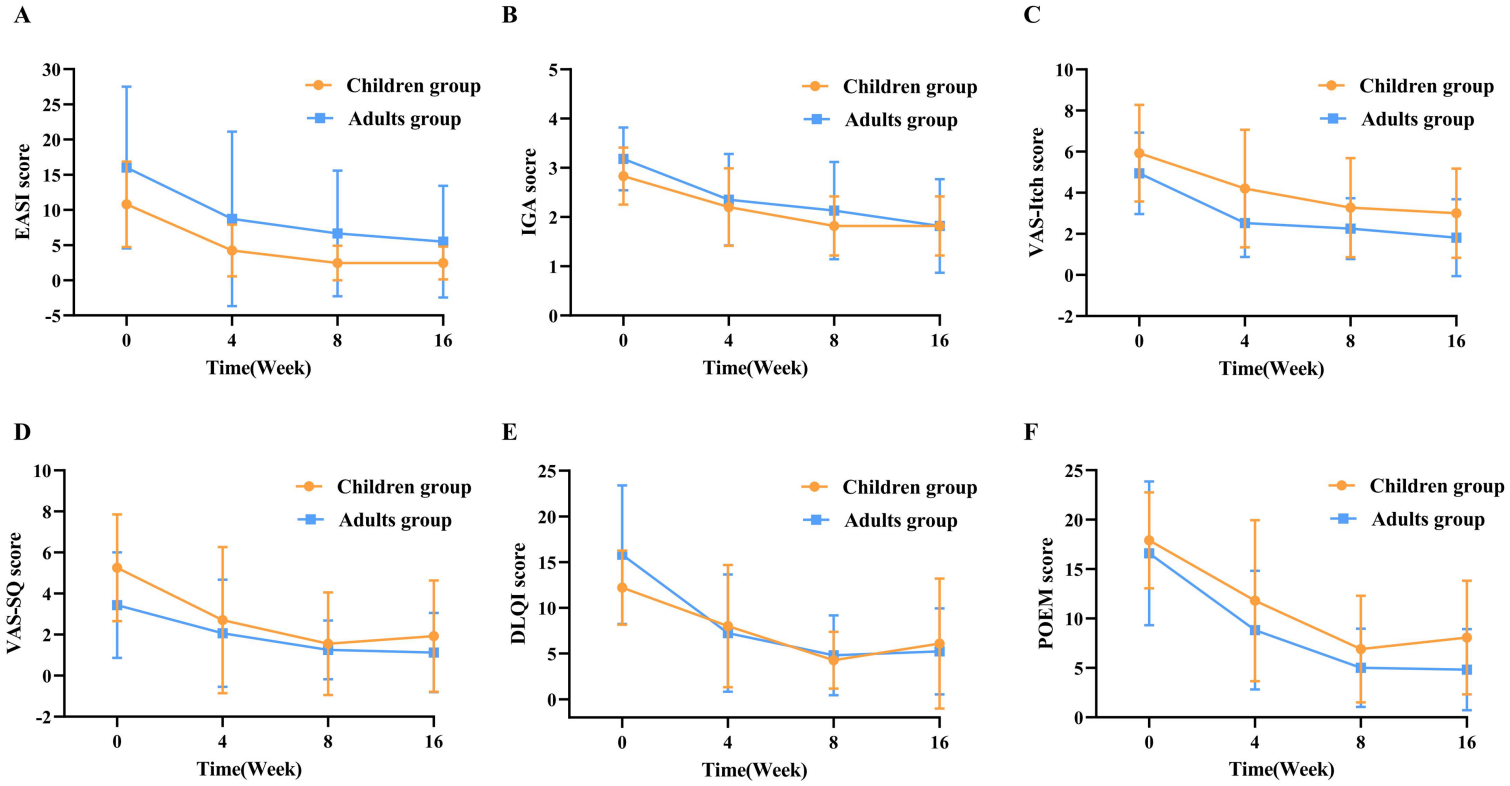
Evolution of mean clinical scores from baseline to week 16. **(A)** Mean scores of EASI at baseline and during follow-up for both groups. **(B)** Mean scores of IGA at baseline and during follow-up for both groups. **(C)** Mean scores of VAS-Itch at baseline and during follow-up for both groups. **(D)** Mean scores of VAS-SQ at baseline and during follow-up for both groups. **(E)** Mean scores of DLQI at baseline and during follow-up for both groups. **(F)** Mean scores of POEM at baseline and during follow-up for both groups.

**Figure 3:**
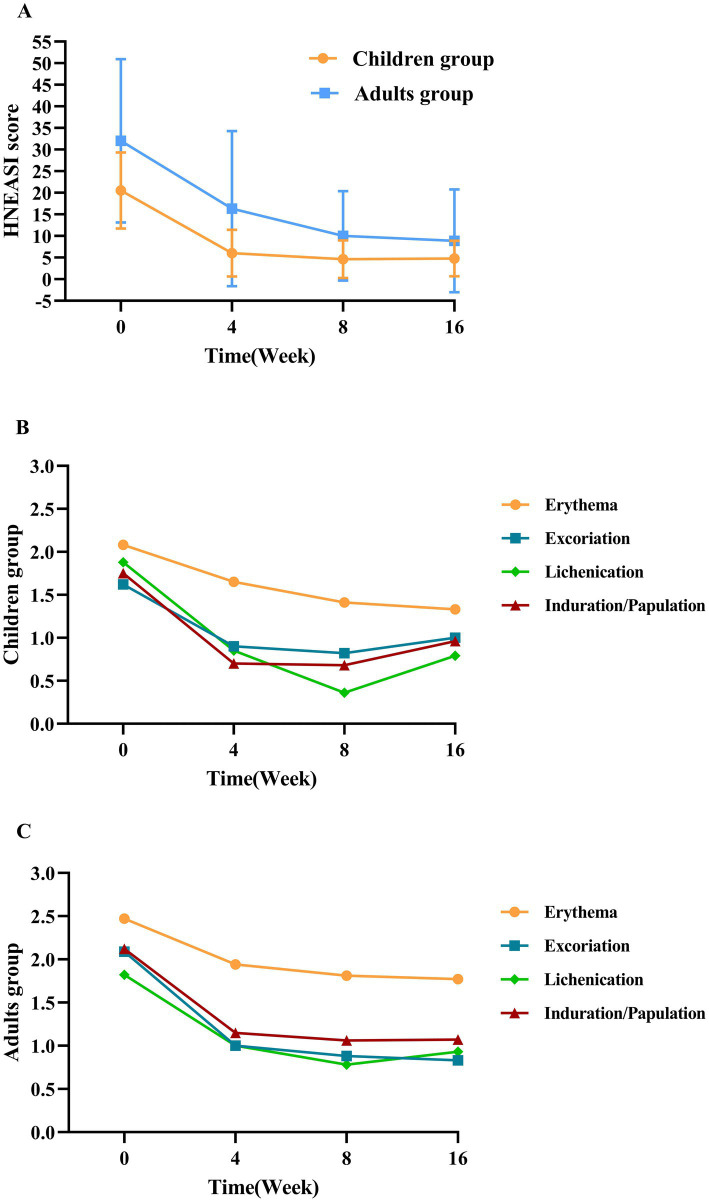
Evolution of mean clinical scores from baseline through week 16. **(A)** Mean total HNEASI scores at baseline and during follow-up for both groups. **(B)** Mean scores of individual HNEASI symptom domains (erythema, excoriation, lichenification, and induration/papulation) at baseline and during follow-up for the pediatric group. **(C)** Mean scores of individual HNEASI symptom domains (erythema, excoriation, lichenification, and induration/papulation) at baseline and during follow-up for the adult group.

At week 4, 70.0% of children achieved EASI-50, and 40.0% achieved EASI-75. By week 16, the proportions achieving EASI-50, EASI-75, and EASI-90 were 75.0, 66.7, and 33.3%, respectively (Figure 1). The EASI score showed a significant reduction from baseline to week 16 (10.80 ± 6.10 vs. 2.45 ± 2.34, *p* < 0.001), accompanied by significant IGA improvement (2.90 ± 0.60 vs. 1.80 ± 0.60, *p* = 0.012) (Figure 2). At week 4, 80.0% of children achieved HNEASI-50, 60.0% achieved HNEASI-75, and 20.0% achieved HNEASI-90. By week 16, the proportions achieving HNEASI-50, HNEASI-75, and HNEASI-90 were 83.3, 75.0, and 16.7%, respectively (Figure 1). The HNEASI score also showed a significant reduction from baseline to week 16, indicating substantial improvement in head and neck symptoms (20.5 ± 8.83 vs. 4.75 ± 4.10, *p* < 0.001). Regarding individual symptom domains of HNEASI scores, erythema, excoriation, lichenification, and induration/papulation all showed significant reductions (2.08 ± 0.63 vs. 1.33 ± 0.72, *p* = 0.040; 1.62 ± 0.88 vs. 1.00 ± 0.67, *p* = 0.044; 1.88 ± 0.48 vs. 0.79 ± 0.75, *p* = 0.002; 1.75 ± 0.87 vs. 0.96 ± 0.81, *p* = 0.037) (Figure 3).

Furthermore, VAS-Itch and VAS-SQ scores significantly decreased at week 16 compared to baseline (5.92 ± 2.35 vs. 3.00 ± 2.17, *p* = 0.002; 5.25 ± 2.60 vs. 1.92 ± 2.71, *p* = 0.002). The DLQI score decreased at week 16, although the change was not statistically significant (12.20 ± 4.10 vs. 6.10 ± 7.10, *p* = 0.079). The POEM score showed a statistically significant reduction (17.90 ± 4.90 vs. 8.10 ± 5.80, *p* = 0.001) (Figure 2).

### 3.2 Adult group

At week 4, 64.7% of adult patients achieved EASI-50, and 41.2% achieved EASI-75. By week 16, the proportions achieving EASI-50, EASI-75, and EASI-90 were 70.6, 52.9, and 23.5%, respectively (Figure 1). A significant reduction in EASI score was observed from baseline to week 16 (16.00 ± 11.50 vs. 5.49 ± 7.95, *p* < 0.001), with IGA improvement (3.20 ± 0.70 vs. 1.80 ± 1.00; *p* < 0.001) (Figure 2). Head and neck rashes demonstrated similar trends, with 58.8% of adult patients achieving HNEASI-50, 41.2% achieving HNEASI-75, and 17.6% achieving HNEASI-90 at week 4. By week 16, the proportions achieving HNEASI-50, HNEASI-75, and HNEASI-90 were 76.5, 64.7, and 29.4%, respectively (Figure 1). The HNEASI score showed a significant reduction from baseline to week 16, indicating substantial improvement in head and neck symptoms (32.0 ± 18.9 vs. 8.88 ± 11.9, *p* < 0.001). Regarding individual symptom domains of HNEASI scores, erythema, excoriation, lichenification, and induration/papulation all showed significant reductions (2.47 ± 0.65 vs. 1.77 ± 0.90, *p* = 0.005; 2.09 ± 1.00 vs. 0.88 ± 0.89, *p* = 0.002; 1.82 ± 0.92 vs. 0.93 ± 0.65, *p* = 0.007; 2.12 ± 0.70 vs. 1.07 ± 0.84, *p* = 0.002) (Figure 3).

VAS-Itch and VAS-SQ scores significantly decreased (4.94 ± 1.98 vs. 1.81 ± 1.87, *p* < 0.001; 3.44 ± 2.58 vs. 1.12 ± 1.93, *p* = 0.009). Both DLQI (15.80 ± 7.60 vs. 5.20 ± 4.70, *p* < 0.001) and POEM (16.60 ± 7.30 vs. 4.80 ± 4.10, *p* < 0.001) demonstrated robust declines (Figure 2).

### 3.3 Safety

During the 16-week follow-up period, no AEs were reported in the adult group. In the children’s group, four patients (33.3%) developed mild conjunctivitis, all of which resolved with symptomatic treatment and did not require treatment discontinuation.

## 4 Discussion

AD is a chronic inflammatory skin disease that significantly impairs the physical, psychological, and social functioning of patients, leading to a decline in quality of life (QoL) (1). This burden not only affects the patients but also their caregivers, resulting in a dual negative impact on QoL. For example, a study involving 265 patients and caregivers found that AD imposed considerable physical, psychological, and economic burdens on the patients, and the patients reported a significant impairment in their QoL (28). Itchiness, the core symptom of AD, has a direct effect on the QoL. In one study, 92.6% of patients ranked relief from itchiness as their most urgent treatment need (29). Moreover, AD can lead to severe sleep disturbances, further exacerbating the burden on the QoL, with itchiness and skin pain being the main contributing factors (30). Research has shown that interventions targeting itchiness and sleep can directly improve QoL (31). In a study of 150 adult AD patients, sleep problems were emphasized as one of the key factors affecting the QoL (32). Therefore, AD management should prioritize symptom control and the improvement of QoL. However, HNAD is notably difficult to manage owing to the limited therapeutic options available in this sensitive area. Dupilumab has emerged as a breakthrough therapy, demonstrating positive efficacy and safety profiles in clinical trials involving patients across different age ranges (12, 33).

This real-world study indicated that dupilumab demonstrated considerable efficiency in both pediatric and adult populations with predominantly head and neck AD, providing short-term relief from itching and a notable reduction in skin lesions by week 16 of therapy.

Our findings align with all previous studies. A *post-hoc* analysis of four phase III studies revealed that dupilumab provided comparable EASI improvements across all anatomical areas in adults, including the upper limbs, lower limbs, trunk, and head and neck (34). In a real-world study, 30.7% of patients with AD achieved complete remission in the head and neck region during the first 4 months of therapy (35). Another study showed that dupilumab induced clinical improvement in skin lesions across various anatomical regions, including the head and neck, in children aged 6 months to 5 years (18). Unlike previous studies that analyzed typical AD without focusing on head and neck lesions, our research provided a real-world assessment of the efficacy of dupilumab specifically in patients with head and neck-dominant AD. Our research demonstrated that 33.3% of pediatric patients and 23.5% of adult patients achieved EASI-90 after 4 months of therapy, consistent with previous findings. Additionally, HNEASI scores showed a significant decline from baseline to week 16. At week 16, 16.7% of children and 29.4% of adults achieved EASI-90, following the same trend observed in the overall EASI scores. However, the proportion was slightly lower in the children’s group than in the adult group, which may be attributed to children’s difficulty in controlling autonomous behaviors, such as repetitive scratching. This finding is consistent with a slight increase observed at week 16 compared to the increase at week 8 in HNEASI subscores for excoriation, lichenification, and induration/papulation (excluding erythema), as well as in VAS-Itch scores.

HNAD significantly negatively affects patient QoL due to lesion localization in exposed areas. Studies indicated that people with moderate-to-severe AD affecting the head, face, neck, and/or hands had much higher deterioration in health-related QoL (36). Multiple previous studies have consistently reported that dupilumab significantly improves QoL in AD (37–39). A recent meta-analysis of randomized controlled trials indicated that dupilumab significantly improves DLQI scores in adult patients and CDLQI scores in pediatric populations (37, 40). Real-world studies have also underscored the robust improvement in QoL associated with dupilumab therapy in routine clinical settings (41–43). Furthermore, studies have observed that patients with persistent or intermittent head and neck lesions after dupilumab treatment maintain elevated DLQI scores, strongly suggesting that therapeutic response in the head and neck region is an important determinant of overall QoL improvement (44). In our study, all assessed clinical scores, including DLQI, VAS-Itch, VAS-SQ, and POEM, exhibited substantial reductions in adult patients with HNAD after 16 weeks of dupilumab therapy, confirming the beneficial impact of dupilumab on the quality of life in these patients (11).

Although dupilumab-associated head and neck dermatitis has been previously reported, no such cases were observed in the present study (22–24). Conjunctivitis is a recognized side effect of dupilumab treatment. During the follow-up period, new-onset conjunctivitis occurred in four children (33.3%) in this cohort, indicating an incidence rate that is considerably higher than the approximately 5–10.3% reported in clinical trials and real-world studies on pediatric patients (12, 17, 45). This discrepancy may be attributable to the inclusion of patients with predominant head and neck lesions in our study, as emerging research suggests that HNAD may constitute a risk factor for dupilumab-associated conjunctivitis (46, 47).

Our study has several limitations. First, the data were collected retrospectively; therefore, we cannot guarantee an entirely timely data collection. Second, the sample size was limited, and the follow-up period was only 16 weeks at the time of data assessment.

In conclusion, our study confirms that dupilumab is effective in both children and adult patients with HNAD. No serious or unexpected adverse events were observed, with only four isolated cases of mild conjunctivitis reported. These findings support the favorable safety profile of dupilumab in HNAD.

## Data Availability

The raw data supporting the conclusions of this article will be made available by the authors, without undue reservation.
